# Characterization of glucose‐stimulated insulin release protocols in african green monkeys (*Chlorocebus aethiops)*

**DOI:** 10.1111/jmp.12374

**Published:** 2018-10-25

**Authors:** Shervin Liddie, Haruka Okamoto, Jesper Gromada, Matthew Lawrence

**Affiliations:** ^1^ RxGen Inc. New Haven Connecticut; ^2^ St. Kitts Biomedical Research Foundation Basseterre St. Kitts W.I; ^3^ Regeneron Pharmaceuticals Inc. Tarrytown New York

**Keywords:** African green monkey, aging, diabetes, graded glucose infusion, insulinopenic, intravenous glucose tolerance test, nonhuman primates, oral glucose tolerance test, plasma insulin, vervet

## Abstract

**Background:**

Management of diabetes remains a major health and economic challenge, demanding test systems in which to develop new therapies. These studies assessed different methodologies for determining glucose tolerance in green monkeys.

**Methods:**

Twenty‐eight African green monkeys between 4 and 24 years old underwent single or repeat intravenous glucose tolerance testing (IVGTT), oral glucose tolerance testing (OGTT), and/or graded glucose infusion testing.

**Results:**

Geriatric monkeys exhibited glucose intolerance with impaired glucose‐stimulated insulin secretion following IVGTT. Repeat IVGTT and OGTT assessments were inconsistent. Monkeys with low glucose‐stimulated insulin secretion after graded glucose infusion exhibited elevated blood glucose levels.

**Conclusion:**

IVGTT and graded glucose infusion protocols revealed differences in glucose tolerance among green monkeys at single time points, including age‐dependent differences suggestive of shifts in pancreatic beta‐cell functional capacity, but care should be applied to study design and the interpretation of data in the setting of longitudinal studies.

## 1 INTRODUCTION

An estimated 30 million Americans were diabetic in 2015, with the percentage of adults with diabetes increasing with age, reaching 25% among those aged 65 years or older.^1^ Diabetes is a leading cause of death in the United States with associated healthcare costs reaching an $245 billion in 2015 alone.^1^ Evaluation of candidate therapeutics to improve management of diabetes remains a critical need to alleviate the health, and social and economic impact.

Type 2 diabetes (T2D), the most common form of diabetes accounting for 90% to 95% of cases,^2^ develops when the pancreatic beta cells do not produce enough insulin to overcome insulin resistance in peripheral tissues involved in glucose absorption, metabolism, and removal. The prevalence of T2D increases with age and often develops during adulthood, although occurrence in older children is becoming more prevalent.^3^ The two primary causes of glucose intolerance in T2D—peripheral insulin resistance and impaired insulin secretion from pancreatic β‐cells—both exhibit change with age.^4^ The primary stimulus for secretion of insulin from β‐cells is circulating glucose, and aging is associated with a marked reduction in glucose‐stimulated insulin release in both rodents and humans.^5^ Apart from advancing age, dyslipidemia characterized by elevated triglycerides and low‐density lipoprotein (LDL) and reduced high‐density lipoprotein (HDL) cholesterol are also associated with T2D^6^ and were additionally assessed in this study.

Nonhuman primates have been extensively used to study the pathology of diabetes as commonly used species, including macaques and African green monkeys, develop spontaneous diabetes, and exhibit many of the characteristic features of diabetes similar to humans, including obesity, insulin resistance, dyslipidemia, and pancreatic pathology.^7^ Many of the physiological factors that predispose humans to T2D, including fluctuation in sex hormone levels, menopause, psychosocial stress, caloric restriction, and early life experiences, also predispose nonhuman primates to T2D.^7^

Several strategies have been developed to aid in (a) the diagnosis of diabetes and (b) the understanding of the progression of diabetes. Variations of the glucose tolerance test are employed clinically to test for diabetes, insulin resistance, impaired beta‐cell function, or other disorders of carbohydrate metabolism.^8^, ^9^ The hyperinsulinemic‐euglycemic clamp method is generally considered the “gold standard” for evaluation of insulin sensitivity, but this test is more invasive, time‐consuming, and costly. Both oral and intravenous glucose tolerance tests, which are less technically involved and similarly informative, have been used extensively in both clinical and preclinical studies to measure how well the body is able to respond to and dispose of glucose, or other dietary sugars. Glucose‐stimulated insulin release can be produced by a single bolus glucose intravenous injection (IVGTT), by a fixed dose intravenous glucose infusion or by orally administered glucose. One limitation of these techniques, however, is that they do not allow for construction of a dose‐response curve between glucose and insulin secretion rates at different glucose concentrations as offered by the graded glucose infusion protocol.^10^ The glucose‐stimulated insulin release protocol to employ will depend on feasibility within larger study design and execution objectives and the specific scientific questions to be answered.

Continued evaluation and interrogation of current methods and models for robustly evaluating glucose tolerance in nonhuman primates with close homology to humans would allow more predictive evaluation of the efficacy of antidiabetic drugs to guide clinical trials. The original impetus for this study was to define cohorts of monkeys that were potentially insulin‐resistant and prediabetic for enrollment in studies to test efficacy of antidiabetic drugs. Based on previous reports, phenotypes that potentially fall within that category would be older, heavier, or dyslipidemic monkeys. The aim of this study was twofold: (a) to characterize three glucose‐stimulated insulin release protocols in African green monkeys and (b) to investigate the effect of age on glucose tolerance.

## 2 METHODS AND MATERIALS

### 2.1 Humane care guidelines

All protocols adhere to The Guide for the Care and Use of Laboratory Animals,^11^ and all studies were approved by the Institutional Animal Care and Use Committee of St. Kitts Biomedical Research Foundation and Regeneron Pharmaceuticals, Inc.

### 2.2 Subjects

Fourteen male and 14 female African green monkeys ranging in age from 4 to 24 years old and weighing from 3.0 to 7.5 kg were enrolled in the study following confirmation of overall good health by physical examination. All monkeys were from the colony at St Kitts Biomedical Research Foundation (SKBRF), St. Kitts, West Indies. Monkeys were either purpose‐bred at the facility or captured humanely from the feral population on the island of St. Kitts and maintained at SKBRF. The age of wild‐caught monkeys was estimated based on general body appearance and dental examination.^12^ Monkeys with known or estimated ages of 14 years and above were considered geriatric. Monkeys were singly housed in stainless steel squeeze cages (32″ × 28″ × 32″) throughout the length of the study and provided water ad libitum. Ambient temperature ranged from 21.6 to 32.6°C over the duration of the study within the ventilated study enclosure. Humidity ranged from 52.5% to 95%. All monkeys were fed pelleted monkey chow biscuits (Harlan Teklad, Madison, Wisconsin, crude protein = 20%, crude fat = 5%, crude fiber = 10%).

### 2.3 Procedures

#### 2.3.1 Phenotypic characterization of geriatric vs young adult cohorts

One of the goals of this study was to identify a cohort of potentially insulin‐resistant and prediabetic monkeys. Phenotypes that fall within that category were expected to be monkeys that were older, heavier, or dyslipidemic. As such, we evaluated biometric parameters and lipid profiles in a cohort of geriatric monkeys and young adult comparators. Evaluation of both biometry parameters and lipid profiles would allow for identification of more significant contributors to potential insulin resistance observed. Monkeys recruited to the study included six colony born, skeletally, and sexually mature males aged 4‐6 years (“young” cohort); six colony born, skeletally, and sexually mature females aged 4‐6 years (“young” cohort); six wild‐caught, geriatric males approximately 14‐20 years old; and six colony born, geriatric females aged 19‐26 years. Wild‐caught geriatric males were fully acclimated to the facility as these monkeys were all trapped and brought to the facility at least 4 years prior to study initiation (four of the six monkeys had been at the facility for greater than 10 years). These monkeys were fed the same diet and maintained in housing conditions identical to all colony born monkeys enrolled in this study. All wild‐caught monkeys were well adjusted to captivity as no overt behavioral differences were observed compared to the colony born monkeys enrolled in this study. Phenotypic data including body weight, crown‐rump length, and girth were measured in the 12 geriatric and 12 young monkeys evaluated in this study. All measurements were performed after overnight fasting and following sedation with ketamine HCl (8 mg/kg, IM; Ketaset^®^, Fort Dodge, IA). Crown‐rump length was measured from the inion to the base of the tail while girth measured at the level of the umbilicus using a flexible tape measure. Additionally, a two‐milliliter sample of blood was collected from the femoral vein using a 20 gauge 1‐inch needle and transferred to a K_2_EDTA‐anticoagulated vacutainers (Becton Dickinson, Rutherford, NJ). The vacutainer was then inverted several times prior to placement on ice for approximately 30 minutes. Blood samples were then centrifuged at 2500×*g* for 7 minutes at 4°C. Two plasma aliquots of approximately 0.5 mL were transferred to appropriately labeled 1.8 cc cryotubes (Nunc, Roskilde, Denmark) and analyzed (Antech Diagnostics, Memphis, TN) for total cholesterol, HDL, LDL, and triglycerides.

To evaluate the differential blood glucose and plasma insulin response of young and geriatric monkeys to a bolus intravenous glucose load, monkeys were subjected to an IVGTT. After an overnight fast (14‐18 hours) achieved by removing all food from the feed pan, cage floor, and anywhere within the monkey's reach, monkeys were sedated by intramuscular administration of ketamine HCl (8 mg/kg; Ketaset^®^) for bolus IVGTT. Booster doses of ketamine (4‐5 mg/kg) were administered as necessary to complete the experiment. Ketamine was selected on the basis of minimal demonstrated effect on glucose levels compared to other anesthetic regimens.^13^, ^14^ General well‐being was assessed before, during and after sedation. Two catheters were placed, one in the arm (cephalic vein) for dextrose injection and one in the leg (saphenous vein) for blood sampling, except on a few occasions in which phlebotomies were performed at the site of the femoral vein when the saphenous vein collapsed as a result of repeat blood draws. Catheter patency was maintained by flushing 0.9% saline into the catheter via a three‐way stopcock during dosing and blood collection. Glucose was administered by intravenous delivery of a 250 mg/kg (0.5 mL/kg) bolus (given over 15 seconds) of 50% dextrose solution followed by a 2 mL saline flush through the catheter. Serial blood samples were collected into heparin anticoagulated blood tubes twice prior to dosing (−5 and 0 minutes) and at 3, 5, 7, 10, 15, 20, and 30 minutes after dosing. We evaluated up to 30 minutes postglucose load because we were particularly interested in the acute insulin response in this cohort of animals.

Blood samples were obtained at each time point by first withdrawing at least 0.4 mL of dead space in the catheter and observing for undiluted blood in the catheter. The syringe was then changed, and a new syringe was used to withdraw the targeted blood volume (~1.0 mL) before transfer to heparinized blood collection tubes for plasma isolation. A slight overage was used for measuring blood glucose concentration in the whole blood sample using a handheld glucometer (Bayer Contour^™^ Ascensia Diabetes Care US, Inc., Parsippany, NJ, USA). The handheld glucometer measurement at each time point represented the average of two measurements obtained one right after the other that agreed within 10% of each other, otherwise a third measurement was performed. For each blood sample time point, the collected blood was first placed into the blood collection tubes, and the remainder in the syringe was used for glucose measurements using the handheld glucometer. Following transfer of blood samples to heparinized tubes, ~20 μL (2 μL per 100 μL blood) of combined dipeptidyl peptidase 4 (DPP IV) and protease inhibitor solution was added, while samples were maintained on ice. DPP IV/protease inhibitor solution was prepared by dissolving mini EDTA‐free protease inhibitor cocktail tablet (Roche Diagnostics, item no. 11836170001, Indianapolis, IN, USA) in 500 μL distilled water and combining with 500 μL DPP IV inhibitor (EMD Millipore Corporation, Burlington, MA, USA item no. DPP4‐010). The blood collection tubes were maintained on ice to prevent the metabolism of glucose by red blood cells in the blood sample. After a blood sample was collected at each time point, the catheter was flushed and the patency maintained by flushing periodically with 0.9% saline solution. After centrifugation of heparinized blood samples at 1300×*g* for 10 minutes at 4°C, the plasma supernatant was transferred to 1.8 mL cryotubes (Nunc) and frozen at −80°C prior to analysis. Plasma insulin concentration was measured using Mercodia Insulin ELISA kits (cat #'s 10‐1113‐01 and 10‐1132‐01) in accordance with the manufacturer's instructions. Each sample was analyzed in duplicate.

#### 2.3.2 Evaluation of the response to repeat intravenous glucose tolerance test in young adult monkeys

To determine whether exposure to previous glucose tolerance tests affects subsequent glucose and insulin responses to glucose loading, monkeys underwent a repeat IVGTT assessment 11 months apart. In assessment 1 (year 1), eight young monkeys (A488, A489, A499, A606, A644 [males] and A590, A613, and A646 [females]) were selected from the group of young monkeys that were previously enrolled in the study to evaluate the effects of age on glucose tolerance. Because four of the previous 12 young monkeys were unavailable due to enrollment in other studies, an additional four naïve monkeys (K653, K769 [males] and K669, K680 [females]) were recruited as replacements. In assessment 2 (year 2), six monkeys (R488, R489, R644, R590, K669, and K680) were selected for a repeat IVGTT assessment. These monkeys were selected because their glucose response in assessment 1 was comparable. Serial blood samples were collected into heparin anticoagulated blood tubes twice prior to dosing (−5 and 0 minutes) and at 5, 10, 15, 20, 30, 40, 60, 90, 120, and 180 minutes after initiation of the intravenous glucose bolus. Monkeys were maintained under sedation until completion of the 60‐minute blood draw, after which the blood collection catheter was removed and animals returned to their home cages. For subsequent phlebotomy time points (90, 120, and 180 minutes), femoral vein phlebotomies were performed. Ketamine (4‐5 mg/kg) was administered prior to the scheduled phlebotomy at 90, 120, and 180 minutes, as necessary. Blood samples were collected, and glucose levels were measured and processed as described above. Plasma insulin was measured as described above.

#### 2.3.3 Evaluation of the graded glucose infusion protocol in young adult monkeys

The graded glucose infusion protocol was evaluated in the same 12 young monkeys described above—eight young monkeys that were previously enrolled in the study to evaluate the effects of age on glucose tolerance and four additional monkeys to replace those that were no longer available for follow‐up evaluation. After overnight fasting and sedation as described above, two catheters were placed, again one in the arm (cephalic vein) for dextrose injection and one in the leg (saphenous vein) for blood sampling, except in those instances where the femoral vein was employed when saphenous access was compromised. A 50% dextrose solution was used during the graded glucose infusion protocol. At 0 minute (Dose 1), dextrose solution was infused at 5 mg/kg/min (0.6 mL/kg/h) for 20 minutes using a NE‐1000 syringe pump (New Era Pump Systems Inc., Farmingdale, NY). Immediately after the 20‐minute blood sample was taken, the rate of glucose infusion was increased to 10 mg/kg/min (1.2 mL/kg/h) for 20 minutes (Dose 2). Immediately after the 40‐minute blood sample was taken, the rate of glucose infusion was increased to 25 mg/kg/min (3 mL/kg/h) for 20 minutes (Dose 3). Serial blood samples were collected twice prior to dosing (−5 and 0 minutes) and at 10, 15, 20, 30, 35, 40, 50, 55, and 60 minutes after initiation of glucose infusion.

#### 2.3.4 Evaluation of oral glucose tolerance testing in young adult monkeys

Oral glucose tolerance testing was performed on five different occasions to test an effective dose of oral dextrose as well as reproducibility of the methodology. The impetus for pursuing OGTT was to establish a glucose tolerance model and testing facility protocol that has direct clinical application as OGTT is routinely used in clinical settings to test for diabetes. During assessment 1, OGTT was performed in the same six monkeys employed in assessment 2 of the repeat IVGTT experiment (R488, R489, R644 [males], R590, K669, K680 [females]). Assessment 1 of OGTT was performed 3 months after the graded glucose infusion procedure. Following placement of saphenous or femoral vein catheter for blood collection, a 1 g/kg dose of a 50% dextrose solution, followed by 5 mL water, was delivered via a nasogastric tube, while the monkey was held in an upright position. A dose of 1 g/kg was initially selected because this dose is comparable to the 75 g dose recommended by the World Health Organization (WHO) for oral glucose tolerance testing in adult humans^15^ (assuming an average adult human weight of ~75 kg). Serial blood samples were collected twice prior to dosing (−5 and 0 minutes) and at 5, 10, 15, 20, 30, 40, 60, 90, 120, and 180 minutes after initiation of the oral dextrose. Monkeys were kept sedated until completion of the 60‐minute blood draw, after which the IV access catheter was removed and monkeys were returned to their home cages. For subsequent phlebotomy time points (90, 120, and 180 minutes), a needle stick at the femoral vein was performed. Ketamine (4‐5 mg/kg) was administered prior to the scheduled phlebotomy at 90, 120, and 180 minutes time points, as necessary. In assessment 2, the same six monkeys were again tested with a dose of 1 g/kg of a 50% dextrose solution 8 months after the first assessment to investigate reproducibility over a sufficient elapsed interval to encompass potential contributors to variability of glucose‐stimulated response, such as seasonal environmental changes. Because the glucose response of the evaluated cohort was substantially reduced during assessment 2, two of the six monkeys (R644 [male] and K680 [female]) were tested with a higher oral glucose dose (3 g/kg dose of a 50% dextrose solution) to determine whether an increased oral glucose dose was necessary to produce the expected blood glucose response (assessment 3). We chose a dose of 3 g/kg based on previous reports using oral doses of 2 and 4 g/kg in cynomolgus monkeys.^16^, ^17^ The higher oral glucose dose, however, did not result in an increased blood glucose response in monkeys previously evaluated, for which reason in assessment 4, two naïve monkeys (A080 and A120 [both young males]) were recruited to the study and were tested first with a 1 g/kg dose of 50% dextrose solution, which was effective during the first assessment in the original cohort, followed 2 months later (assessment 5) by a 10‐fold increase in the original oral glucose dose (10 g/kg dose of a supersaturated (195%) dextrose solution). The supersaturated solution was formulated using the Sigma product D‐(+)‐Glucose (BioXtra, product code G7528).

### 2.4 Data analysis

Means, standard deviation, and standard error of the mean were calculated where appropriate. Body mass index (BMI) was calculated as monkey weight measured in kilograms divided by crown‐rump length measured in meters squared. One‐way ANOVA with post hoc Tukey's multiple comparisons test was used to compare differences in biometric measures and lipid profiles between geriatric males and female and young male and female monkeys. Student's *t* test was used to compare geriatric monkeys as a group vs young monkeys as a group. A two‐way ANOVA was used to evaluate differences in glucose and insulin responses related to age and time postglucose load in the geriatric vs young IVGTT comparison. A two‐way ANOVA was also performed to evaluate differences in glucose and insulin response in the young monkey cohort at the initial IVGTT assessment and repeat assessment 8 months later. Post hoc comparisons were determined using Tukey's multiple comparisons test. The glucose tolerance index (*K*_G_), which provides an estimate of the rate of glucose elimination after a glucose dose,^18^ was calculated from the slope of the regression line obtained from the plot of the natural logarithm‐transformed glucose data between 3 and 30 minutes, or between 5 and 40 minutes, expressed as a percentage per minute.

To correct for the effect of basal levels of insulin on postglucose load insulin response, peak insulin was calculated as response at 5 minutes postglucose load minus basal insulin levels. To better understand the sensitivity to and response of pancreatic β‐cells to glucose stimulus, as indicated by the first‐phase insulin response, peak insulin area under the curve (AUC) corrected for basal levels was calculated as insulin AUC between 5 and 15 minutes postglucose load minus basal insulin levels. Paired *t* tests were used to compare differences in peak insulin and peak insulin AUC responses between IVGTT tests performed in year 1 vs year 2 in the same monkeys. Additionally, unpaired Student's *t* test was used to evaluate differences in glucose and insulin AUC where appropriate. AUC was calculated using the trapezoid rule. Pearson correlation was used to evaluate the linear relationship between age and either blood glucose or plasma insulin concentrations. Statistical analyses were performed using Prism 6 (GraphPad Software, Inc., La Jolla, CA, USA). Differences between the means were considered significant at *P* < 0.05.

## 3 RESULTS

### 3.1 Comparison of biometric and lipid profiles between geriatric and young adult monkeys

Mean biometry measurements and plasma lipid profile data are presented in Table 1, while individual animal data are presented in Table S1. As expected, there was an overall significant difference in body weight (*F*_(3,20) _= 12.93; *P* < 0.001) and crown‐rump length (*F*_(3,20)_ = 12.97; *P* < 0.001) between sexes. Post hoc analyses showed both geriatric and young males were significantly heavier and longer than geriatric and young females. However, no significant difference in body weight or crown‐rump length was observed between geriatric males and young males or between geriatric females and young females. No significant differences in girth and BMI were observed when all four groups were compared. Similarly, no significant differences in LDL or total cholesterol were observed. Levels of LDL and triglycerides, however, were significantly elevated in geriatric females compared to young males (*P* < 0.05 in both instances). Importantly, there were no differences in lipid profiles between geriatric males (which were all wild‐caught monkeys acclimated to captivity within the facility for at least 4 years) and geriatric females (which were all colony born) or between young males and young females; and geriatric monkeys were grouped together, and young monkeys were grouped together to assess age effects. Triglycerides (*t* = 2.3; df = 22; *P* = 0.0313) and LDL cholesterol (*t* = 2.183; df = 22; *P* = 0.0400) were both significantly elevated in geriatric compared to young monkeys.

**Table 1:** Mean baseline phenotypic data for young and geriatric cohorts

Parameter	Units	Geriatric females		Geriatric males		Young females		Young males		Student's *t* test (*P* value)
		Mean	Stdev	Mean	Stdev	Mean	Stdev	Mean	Stdev	(Geriatric vs Young)
Age	Years	22.7	26	16.3	8.1	4.7	0.8	5.3	1.0	**6.77E‐13**
Weight	kg	4.1	4.716	5.2	2.1	4.3	0.3	6.3	1.1	0.0503
Girth	cm	35.8	40	31.6	13.9	31.9	2.8	35.9	3.9	0.7871
Crown‐rump length	cm	40.5	42	38.3	18.0	39.8	2.3	45.9	1.6	0.0838
BMI	kg/m^2^	25.0	28	24.3	10.5	27.5	3.7	29.8	3.1	0.1269
Cholesterol	mg/dL	149.5	155	120.4	44.2	133.3	16.5	133.0	34.3	0.4714
HDL	mg/dL	56.8	56	58.2	21.4	65.8	11.8	74.5	16.2	0.1587
LDL	mg/dL	91.5	102	60.5	28.6	64.8	17.0	49.0	9.9	**0.0313**
Triglycerides	mg/dL	65.8	40	36.3	17.0	39.3	9.7	27.8	5.9	**0.04**

BMI, body mass index; HDL, high‐density lipoprotein; LDL, low‐density lipoprotein. Bold values, significant differences between geriatric and young at *p* < 0.05

### 3.2 Effect of age on glucose clearance following bolus IVGTT

**Figure 1:**
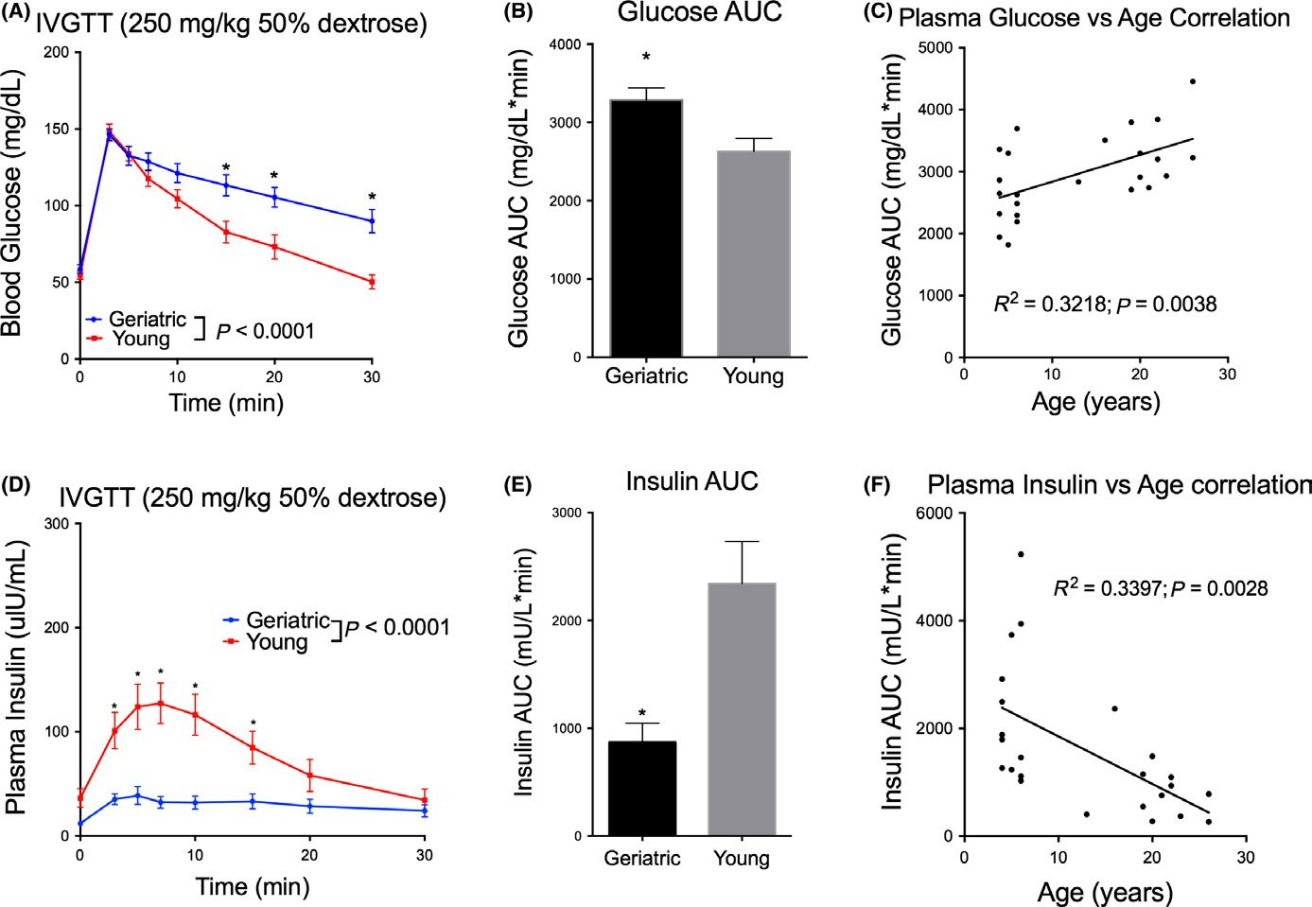
Effect of age on glucose tolerance and glucose‐stimulated insulin release. A, Baseline glucose and peak blood glucose following glucose loading (250 mg/kg of 50% glucose solution) were similar between young and geriatric monkeys. Glucose clearance, however, was significantly faster in young monkeys compared to geriatrics. B, Blood glucose AUC was significantly higher in geriatric compared to young monkeys. C, A significant positive correlation between blood glucose AUC and age was observed. D, Baseline insulin and peak plasma insulin concentration following glucose loading were higher in young compared to geriatric monkeys. Glucose‐stimulated insulin release was suppressed in geriatric monkeys. E, Plasma insulin AUC was significantly higher in young adult monkeys compared to geriatrics. F, A significant negative correlation between plasma insulin AUC and age was observed. Data are presented as mean ± SEM. n = 12/group. *P < 0.05

Blood glucose and plasma insulin concentrations were measured in individual geriatric and young monkeys following bolus injection of 250 mg/kg dextrose at various time points postinjection (Figure 1). Geriatric monkeys displayed slower glucose clearance kinetics than young monkeys (Figure 1A). Comparison of young and geriatric IVGTT data by two‐way ANOVA revealed a significant age group effect [*F*_(1,171) = _31.57; *P* < 0.0001], where the rate of glucose clearance was reduced in geriatric monkeys. Additionally, post hoc analyses showed significantly delayed glucose clearance at 15, 20, and 30 minutes postdextrose injection in geriatric compared to young monkeys. While blood glucose levels returned to baseline within 30 minutes of dextrose loading in young adults, blood glucose levels in geriatric monkeys remained significantly elevated up to 30 minutes postdextrose administration, suggesting delayed glucose clearance in geriatric monkeys (Figure 1A). Glucose tolerance was additionally assessed by comparing the glucose AUC. Geriatric monkeys exhibited decreased glucose tolerance compared to young animals (Figure 1B). Following dextrose injection, young monkeys had a significantly lower blood glucose AUC compared to geriatric animals (unpaired Student's *t* test: *t* = 2.9, df = 22; *P* = 0083). Indeed, geriatric monkeys displayed reduced glucose tolerance index (K_G_) calculated between 3 and 30 minutes (*F*_(1,183)_ = 9.07447; *P* = 0.003) compared to young animals. A significant positive correlation between age and blood glucose concentration was observed (*R* ^2^ = 0.3218; *P* = 0.0038; Figure 1C).

While plasma insulin concentrations increased in response to intravenous glucose loading in young monkeys, insulin levels remained low in geriatric monkeys, suggesting that intravenous glucose administration did not evoke a robust insulin response in geriatric monkeys (Figure 1D). Comparison of young and old IVGTT data by two‐way ANOVA revealed there was a significant age group effect (*F*_(1,168)_ = 75.66; *P* < 0.0001), where overall insulin response was significantly reduced in geriatric monkeys. Post hoc analyses showed significantly elevated insulin levels at 3, 5, 7, 10, and 15 minutes after dextrose injection in geriatric compared to young animals. Additionally, Student's unpaired *t* test showed that following dextrose injection, young monkeys had a significantly higher plasma insulin AUC compared to geriatric monkeys (*t* = 3.44, df = 22; *P* = 0023; Figure 1E). Age was negatively correlated with plasma insulin concentrations (*R* ^2^ = 0.3397; *P* = 0.00328; Figure 1F). See Figure S1 for individual animal blood glucose and plasma insulin response to IVGTT.

### 3.3 Repeat bolus IVGTT

**Figure 2:**
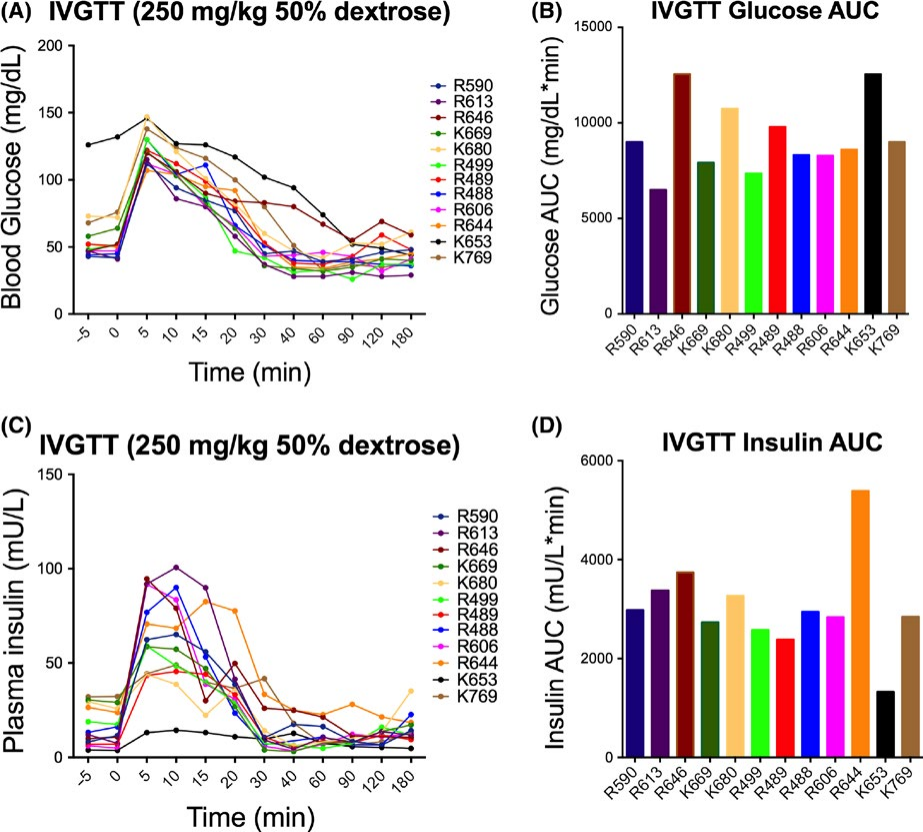
Blood glucose and plasma insulin response following IVGTT. A, Blood glucose values in individual monkeys up to 3 h after glucose load. Monkey K653 was likely diabetic with elevated basal blood glucose. B, Individual monkey glucose AUC up to 3 h after glucose administration. C, Plasma insulin values in individual monkeys up to 3 h after glucose load. Monkey K653 showed suppressed glucose‐stimulated insulin response. D, Individual animal insulin AUC up to 3 h after glucose administration

**Figure 3:**
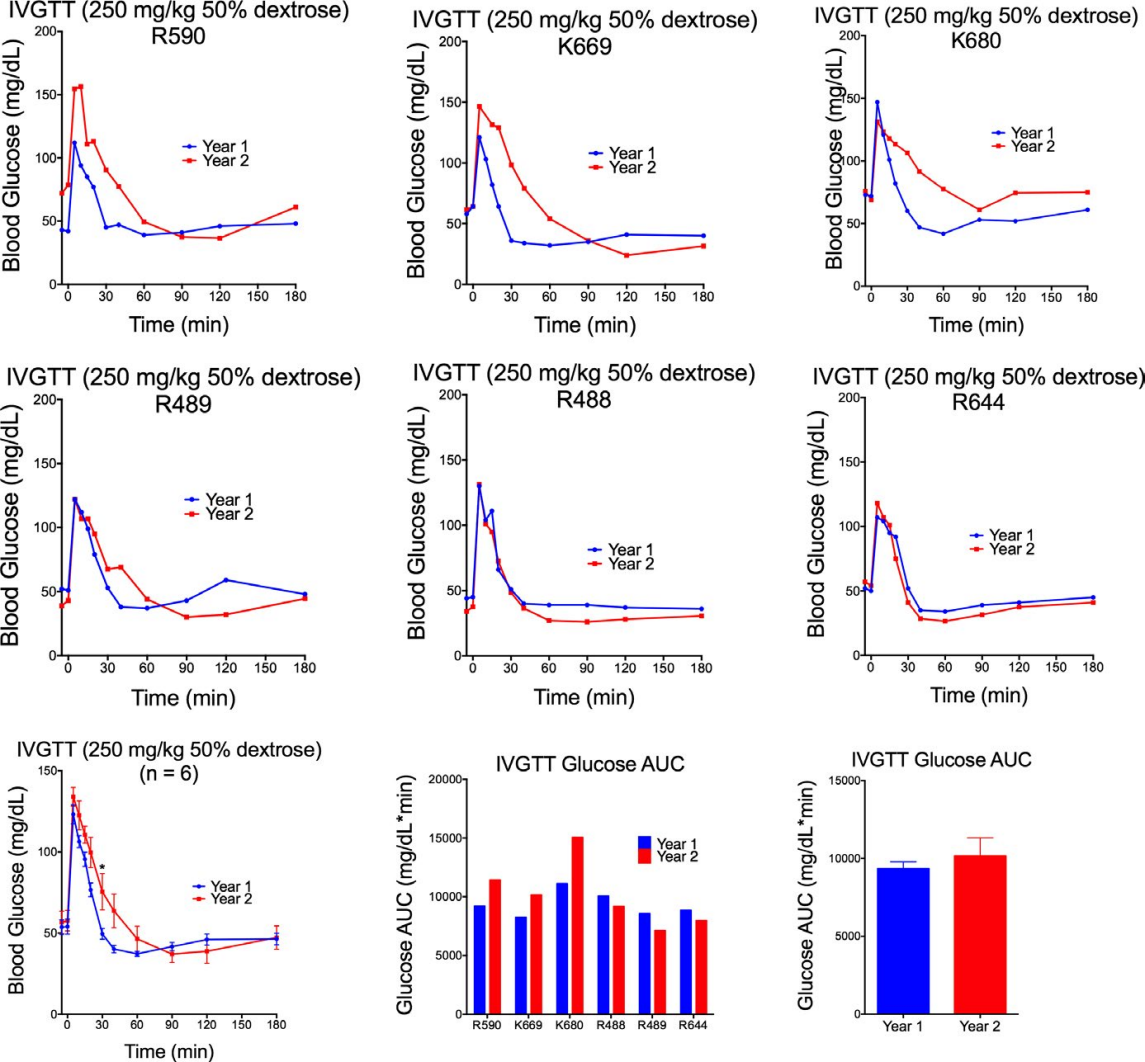
Blood glucose response following repeat IVGTT. Intra‐animal comparison of blood glucose concentration following intravenous administration of 250 mg/kg of 50% dextrose performed in year 1 and year 2 (11 mo apart) for six selected monkeys. Responses in individual monkeys are presented in the top two rows. Group mean responses are presented in the bottom row. At assessment 1 (2015), blood glucose clearance was slightly delayed. *P < 0.05

**Figure 4:**
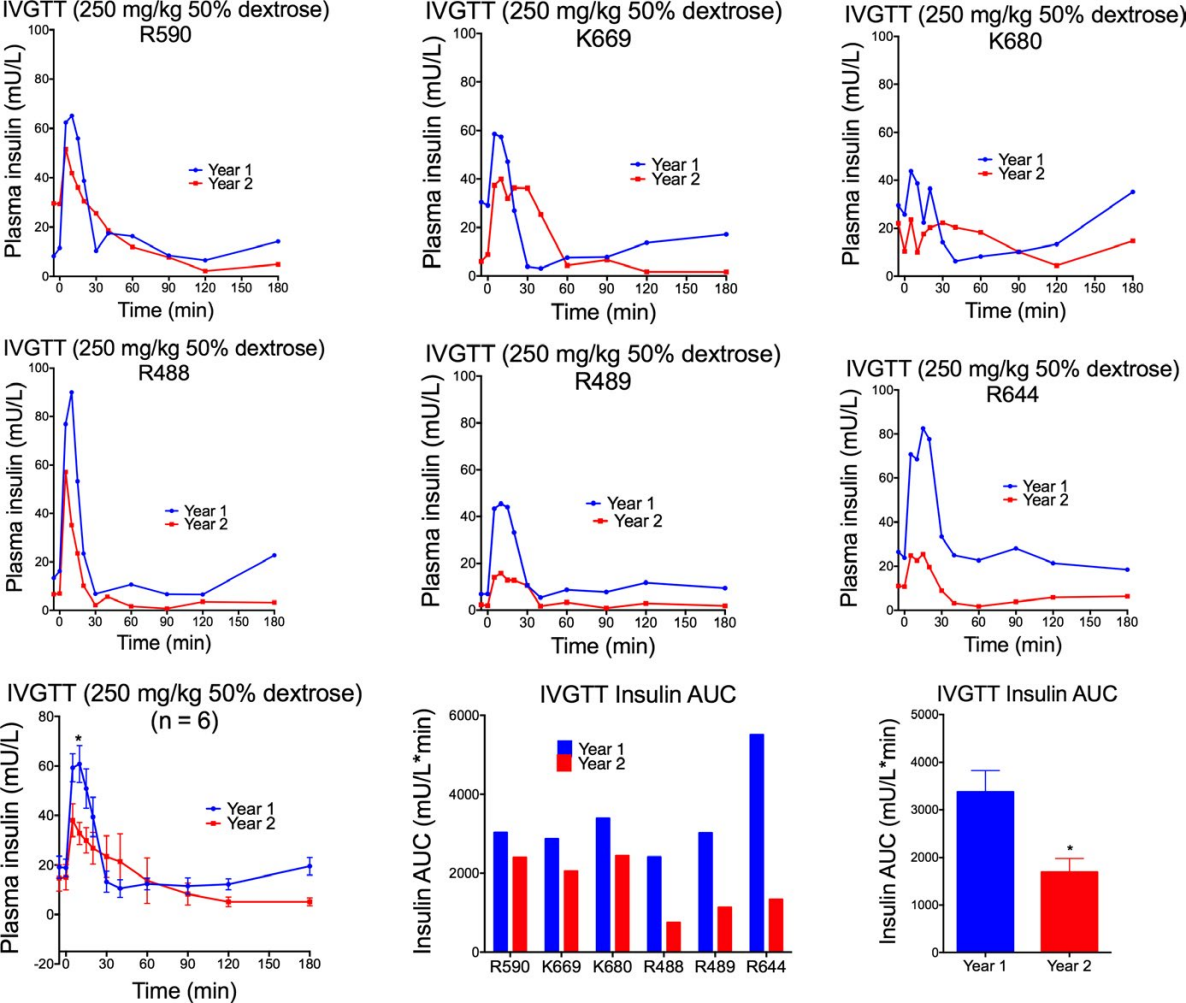
Plasma insulin response following repeat IVGTT. Intra‐animal comparison of plasma insulin concentration following intravenous administration of 250 mg/kg of 50% dextrose performed during year 1 and year 2 (11 mo apart) for six selected monkeys. Responses in individual monkeys are presented in the top two rows. Group mean responses are presented in the bottom row. Peak insulin concentration and insulin AUC were significantly lower during the second test compared to the first test. *P < 0.05

In response to intravenous administration of 250 mg/kg of 50% dextrose, peak blood glucose and insulin concentrations were observed 5 minutes postdextrose infusion and returned to baseline approximately 30 minutes postdose (Figure 2). Monkey K653 (female) displayed a significantly reduced glucose‐stimulated insulin response following IVGTT. Indeed, this monkey also had elevated basal blood glucose levels (Figure 2), suggestive of diabetes. No overall sex differences in blood glucose and plasma insulin response were observed (Figure S2). When comparing assessment 1 (year 1) and assessment 2 (year 2), there was a significant difference in blood glucose response (*F*_(1,120)_ = 15.53; *P* = 0.0001), where the rate of glucose clearance was reduced in year 2 and a significant reduction in the overall insulin response during year 2 (*F*_(1,120)_ = 10.78; *P* = 0.0013; Figure 3). While blood glucose AUC_(0‐180)_ values were similar between the two tests (Figure 3), peak insulin concentrations postglucose infusion and insulin AUC_(0‐180)_, were significantly reduced in year 2 compared to year 1 (AUC: *t* = 3.151, df = 5; *P* = 0.0254) for all six monkeys (Figure 4). The glucose tolerance index K_G_ between 5 and 40 minutes was not significantly different between the two tests. While peak blood glucose concentrations were similar between tests, peak plasma insulin concentration (measured at 5 minutes postdose) was significantly higher (*t* = 2.432; df = 10; *P* = 0.0353) during the first test compared to the second test 11 months later (Figure 4). Additionally, when corrected for basal insulin levels, peak insulin at 5 minutes postglucose load (*t* = 3.020; df = 5; *P* = 0.0294; paired *t* test) and peak insulin AUC (*t* = 5.295; df = 5; *P* = 0.0032; paired *t* test) calculated between 5 and 15 minutes (AUC_(5‐15)_ – basal) were both significantly higher during year 1 compared to year 2 (Figure 4).

### 3.4 Graded glucose infusion

**Figure 5:**
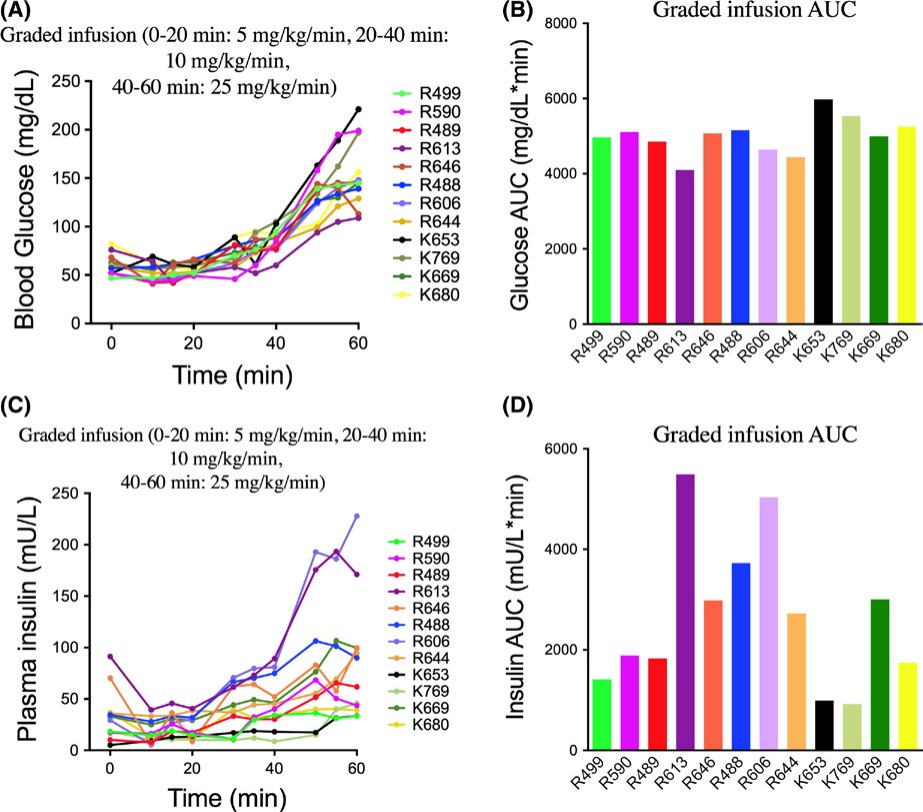
Blood glucose and plasma insulin response following 60‐min graded glucose infusion. Blood glucose (A,B) and plasma insulin (C,D) concentration for individual monkeys following graded glucose infusion of 5 mg/kg/min for 20 min followed by 10 mg/kg/min for 20 min followed by 25 mg/kg/min for 20 min of 50% dextrose. The left panels indicate blood glucose levels up to 60 min postdextrose infusion. The right panels indicate calculated glucose AUC

Twelve monkeys underwent graded glucose infusion testing. Blood glucose levels at the 60‐minute time point ranged from 109 to 221 mg/dL with a mean of 154 mg/dL and a standard deviation of 37 mg/dL (Figure 5A). Blood glucose AUC ranged from 4103 to 5978 mg/dL*min with a mean of 5009 mg/dL*min and a standard deviation of 488 mg/dL*min (Figure 5B). More variability was observed in the insulin response with plasma insulin levels at the 60‐minute time point ranged from 33 to 228 mU/L with a mean of 87 mU/L and a standard deviation of 60 mU/L (Figure 5C). Plasma insulin AUC ranged from 927 to 5463 mg/dL*min with a mean of 2646 mU/L*min and a standard deviation of 1487 mU/L*min (Figure 5D). No significant sex differences in glucose or insulin response to graded glucose infusion were observed (Figure S3). Three monkeys (K653, K769, and R499) displayed reduced glucose‐stimulated insulin responses following the graded glucose infusion protocol, suggesting that these nongeriatric monkeys may be afflicted with insulinopenic diabetes mellitus.

### 3.5 Oral glucose tolerance testing

**Figure 6:**
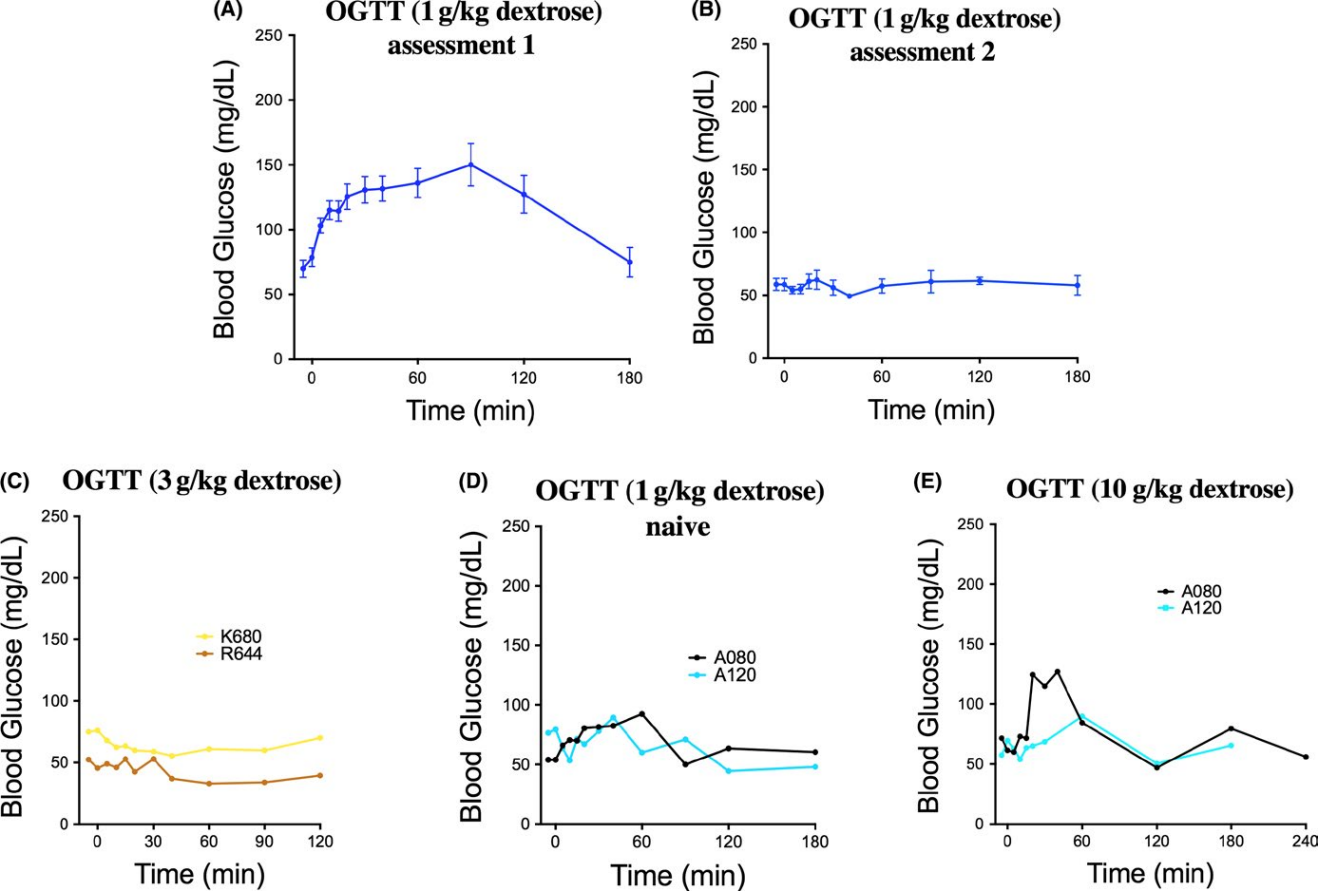
Effect of orally administered glucose on circulating blood glucose concentration. A, Mean blood glucose response following oral administration of 1 g/kg dextrose solution during assessment 1. B, Mean blood glucose response following repeat oral administration of 1 g/kg dextrose solution during assessment 2. For (A and B), data are presented as mean response ± SEM. C, Blood glucose response of two monkeys following repeat OGTT with threefold increase in oral dextrose solution administration (3 g/kg). D, Blood glucose response of two naïve monkeys following single OGTT assessment with oral administration of 1 g/kg dextrose solution. E, Blood glucose response following repeat OGTT with 10‐fold increase in oral dextrose solution administration (10 g/kg)

Oral glucose tolerance testing was highly variable. Individual blood glucose values for the six monkeys that underwent OGTT in assessment 1 are presented in Figure S4A with corresponding AUC values presented in Figure S4B. As a group, peak blood glucose concentration was observed at 90 minutes postdose. At this time point, the mean increase in blood glucose was ~76 mg/dL (Figure 6A). A repeat OGTT assessment was performed 8 months after assessment 1 to determine reproducibility of the OGTT methodology. Mean blood glucose levels remained at or around baseline levels throughout the test (Figure 6B) and calculated glucose AUC fell about 50% compared to assessment 1 (Figure S4). Due to the lack of response to 1 g/kg dextrose in assessment 2, the dose was increased to 3 g/kg to determine whether this would produce a more robust blood glucose response. Two of the original six monkeys were dosed with 3 g/kg dose of dextrose and blood glucose levels in these two monkeys remained at or below baseline throughout the test (Figure 6C).

As OGTT had not produced significantly elevated blood glucose levels even with a 3× increased dose, it was hypothesized that perhaps the monkeys may have developed a compensatory response due to repeated glucose challenges. As such, two naïve monkeys were recruited to the study. These monkeys were tested with an initial dose of 1 g/kg of a 50% dextrose solution (Figure 6D) and subsequently with a dose of 10 g/kg of a supersaturated dextrose solution (Figure 6E). The expected increase in blood glucose following dextrose dosing was not observed at either dose tested. A transient increase in blood glucose was observed 20 minutes after the dose for monkey A080, which was likely due to the fact that the monkey vomited approximately 2 minutes prior to the 20‐minute phlebotomy time point. Emesis of the oral dextrose dose was common, occurring in approximately 60% of cases, during OGTT sessions. These results suggest that OGTT under ketamine‐induced anesthesia, using the experimental conditions we employed, is not a reproducible method for detecting postloading blood glucose levels.

## 4 DISCUSSION

In the present study, we found that geriatric monkeys demonstrate glucose intolerance suggestive of insulinopenic diabetes mellitus. We further found that blood glucose and plasma insulin responses are variable between monkeys and among monkeys during follow‐up IVGTT testing, which should be considered in adequately powering studies and conceiving overall study designs. Additionally, we found that graded glucose infusion presents an alternative test for assessing glucose‐stimulated insulin release and the identification and evaluation of insulin‐resistant monkeys.

Glucose intolerance in geriatric monkeys, indicated by elevated blood glucose levels up to 30 minutes after glucose loading and the lack of insulin response to bolus IVGTT, is consistent with the age‐dependent increase in glucose intolerance and T2D in humans.^5^, ^19^ Although the geriatric males enrolled in this study were all wild‐caught, they had been housed at the facility for at least 4 years. Thus, these monkeys were fully acclimated to the testing facility and were exposed to the same diets and housing environment as the colony born monkeys enrolled in the study. This reduced but did not eliminate the possible confounds of place of birth, as we acknowledge epigenetic influences of early years of life in the wild may have persisting influences on metabolism.^20^ The age‐associated impairment in glucose‐stimulated insulin secretion has been well documented in other species and is likely associated with progressing pancreatic beta‐cell dysfunction.^21^, ^22^ A diminished glucose‐stimulated insulin secretion likely contributed to the impaired glucose tolerance in geriatric monkeys. Additionally, impaired glucose‐stimulated insulin release was accompanied by elevated triglycerides and LDL levels in geriatric monkeys as previously observed,^6^ suggesting that geriatric African green monkeys represent a useful test system in diabetes research.

Glucose‐stimulated plasma insulin response to two separate IVGTT assessments conducted 11 months apart exhibited reduced first‐phase insulin response during the second test compared to the first test. Others have reported increased first‐phase insulin response following two sequential IVGTTs performed within 6 months in humans, possibly resulting from changes in beta‐cell insulin secretion,^9^ while some have reported more consistent insulin response in humans when IVGTT sessions were separated by at least 2 weeks,^23^ or otherwise variable results in humans when two IVGTT sessions were performed within 1 week.^24^, ^25^ A number of factors could contribute to these different findings, including elapsed time between the first and second test,^26^ stress,^27^ species, or loading dextrose dose. A mean reduction in insulin response during the second test may have been responsible for the slightly prolonged blood glucose elevation following the glucose load. Variations in the physiological state of the animals after 11 months may, in turn, have contributed to differential intra‐animal responses to IVGTT, reflective of the physiological state. It has been demonstrated, for instance, that insulin extraction by the liver can vary in response to circulating hormones.^28^, ^29^ Given longitudinal variability in measured glucose‐stimulated first‐phase insulin secretion, care should be taken when interpreting IVGTT data, especially in long‐term studies.

The graded glucose infusion protocol represents a useful alternative to IVGTT for assessing glucose responsiveness of beta cells in individuals with T2D,^30^ applicable to the nonhuman primate. The graded glucose infusion protocol offers similar capabilities as IVGTT and OGTT, but is particularly advantageous for challenging the β‐cell insulin secretory function in response to progressively increased stimulation of β‐cells, which cannot be achieved using single bolus dose or single dose infusion protocols such as IVGTT or OGTT.^10^ Three monkeys demonstrated impaired incremental increases in plasma insulin with exaggerated peak glucose suggestive of possible beta‐cell dysfunction. These data are consistent with the reduced incremental insulin response following graded glucose infusion observed in patients with T2D.^30^

Characterization of blood glucose and plasma insulin response to oral glucose tolerance testing under ketamine sedation proved challenging with regard to reproducibility of the blood glucose responses. The high incidence of emesis may have contributed to the blunted blood glucose response and variability. Subsequent studies will evaluate the use of antiemetic drugs such as chlorpromazine as it has been shown that low doses of the drug do not affect glucose tolerance or glucose‐stimulated insulin release.^31^ Throughout the course of OGTT testing, the following potential confounds were ruled out: (a) dextrose volume and dose, (b) dextrose source, (c) sedation regimen, (d) fasting interval, and (e) effect of repeat testing. One possible confound on response to OGTT in this study is the use of ketamine as a sedative. It has previously been demonstrated in two species of macaques that ketamine might invalidate oral carbohydrate tolerance tests by delaying gastric emptying.^32^ Given that physical restraint has been shown to affect the response to glucose challenge,^33^ these collective findings suggest that definitive evaluation of reproducibility of OGTT response in nonhuman primates may warrant further method development.

It is important to note that a relatively limited number of monkeys were utilized in these studies to assess feasibility and reproducibility of the glucose stimulation protocols explored. Additional investigations using an increased number of monkeys would be warranted to fully validate these findings. Additionally, the length of time between the follow‐up IVGTT and OGTT experiments, with the possible introduction of both biological and technical drift, may have contributed to the limited degree of reproducibility in the response observed. At our testing facility, monkeys are housed in semi‐outdoor enclosures for enrichment purposes. As such, environmental seasonality may affect physiological responses more detectably than might be the case in environmentally controlled indoor facility settings. Such environmental influences should be considered in the interpretation of metabolic data.

In summary, our results show that geriatric monkeys are glucose intolerant relative to younger monkeys, likely resulting from a suppressed glucose‐stimulated insulin response. While the IVGTT test is capable of differentiating monkeys with different degrees of insulin dysregulation in cross‐sectional study designs, variability over time may be observed. Additionally, we show that the graded glucose infusion protocol may represent a useful alternative for assessment of insulin resistance and an additional model in the preclinical setting for the evaluation of insulinotropic therapeutics for combatting T2D.
