# Supplementary figures and images for: Characterization of glucose‐stimulated insulin release protocols in african green monkeys (Chlorocebus aethiops)
Source: J Med Primatol. 2018 Oct 25;48(1):10–21. doi: 10.1111/jmp.12374 (PMC6587791; doi:10.1111/jmp.12374)

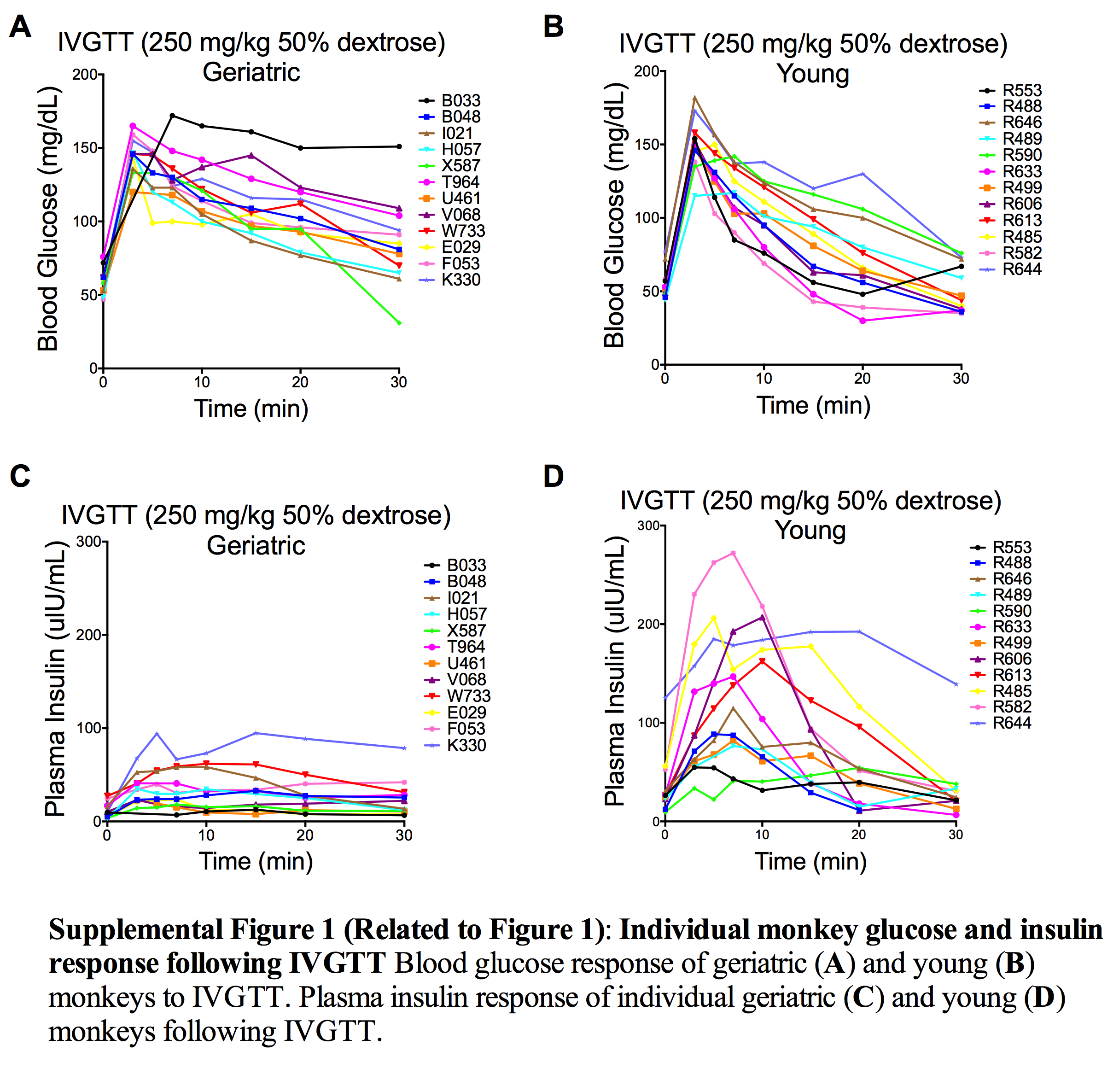

Supplement: Supplementary file 1 [file JMP-48-10-s001.tiff]

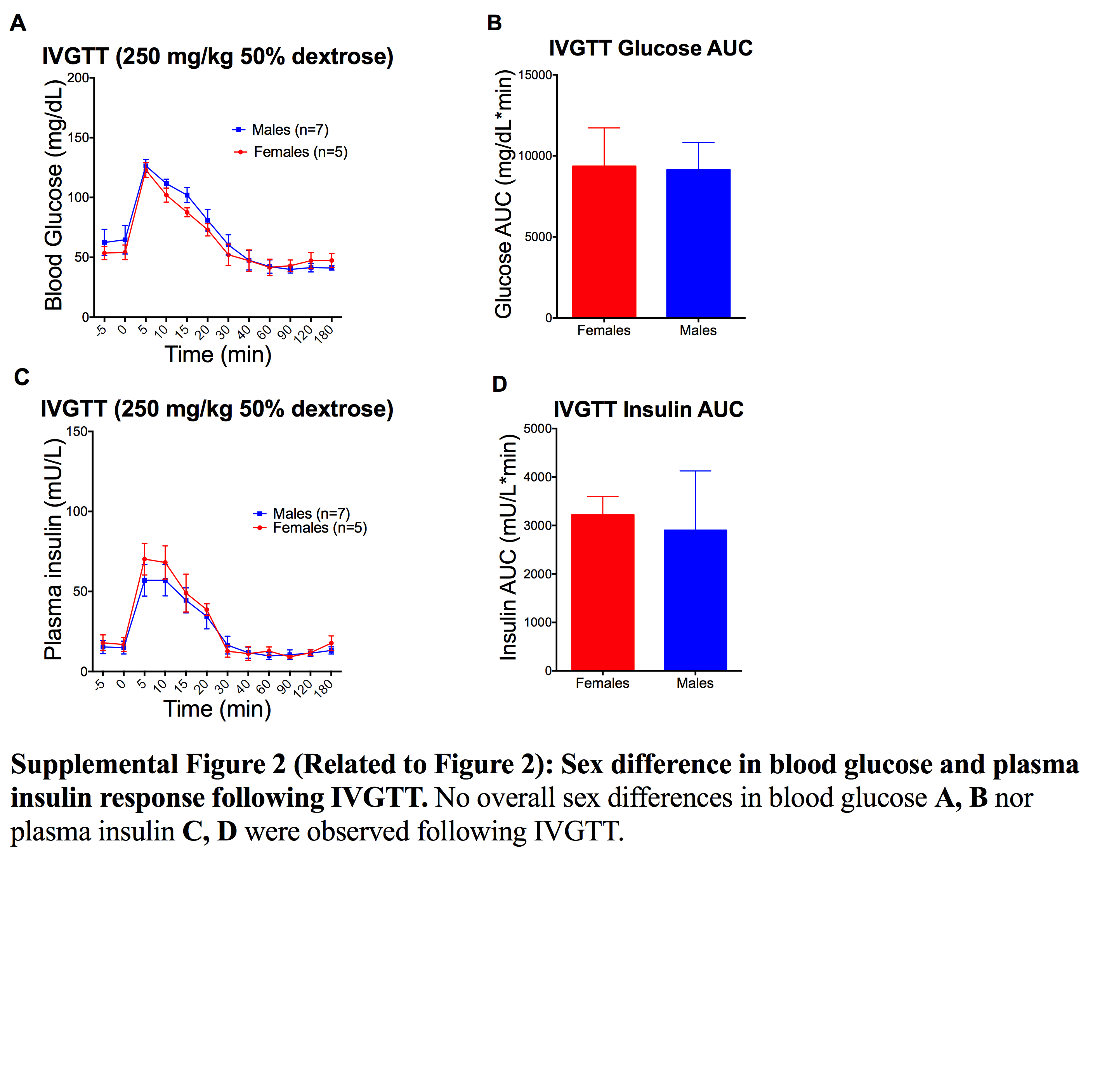

Supplement: Supplementary file 2 [file JMP-48-10-s002.tiff]

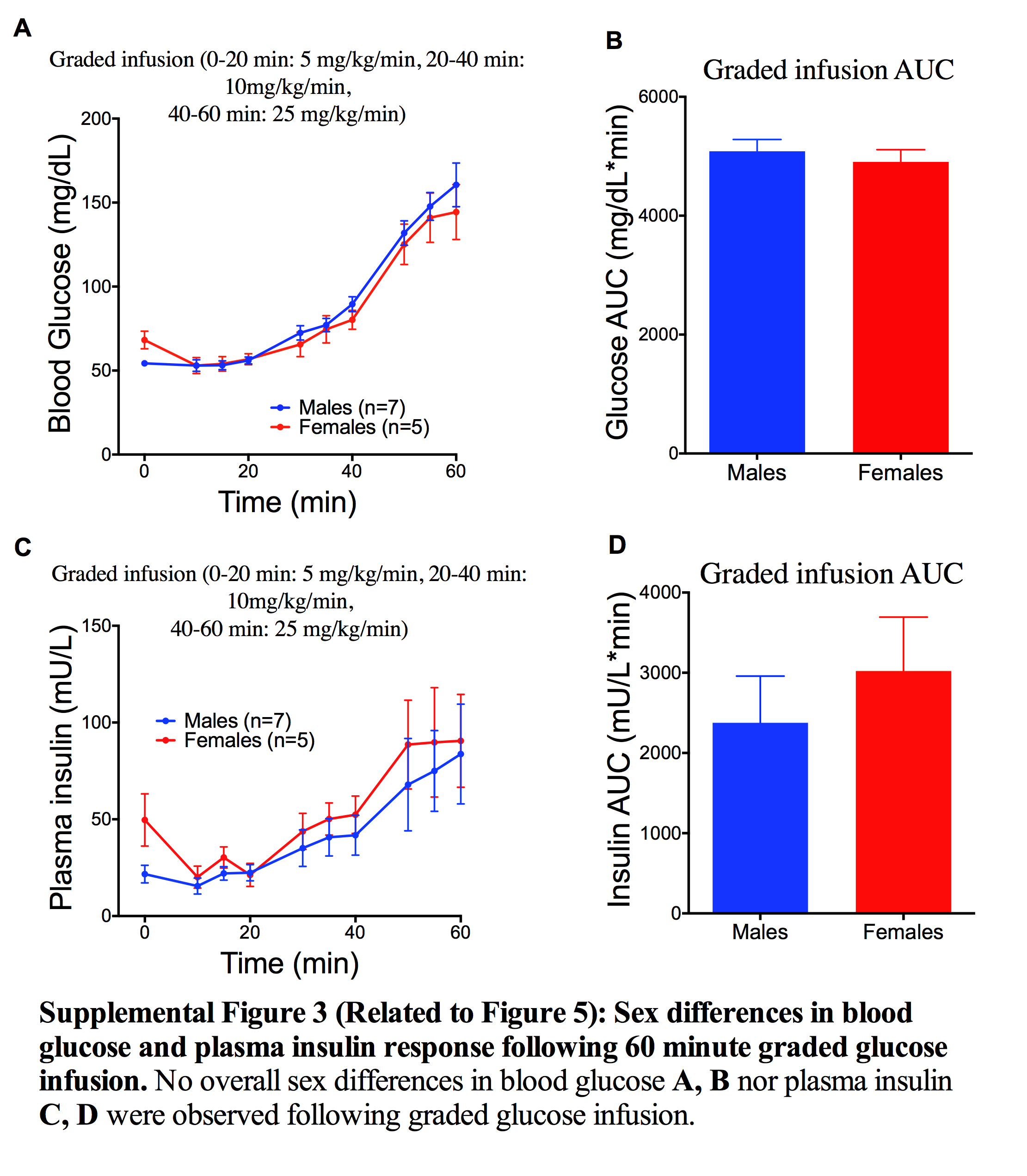

Supplement: Supplementary file 3 [file JMP-48-10-s003.tiff]

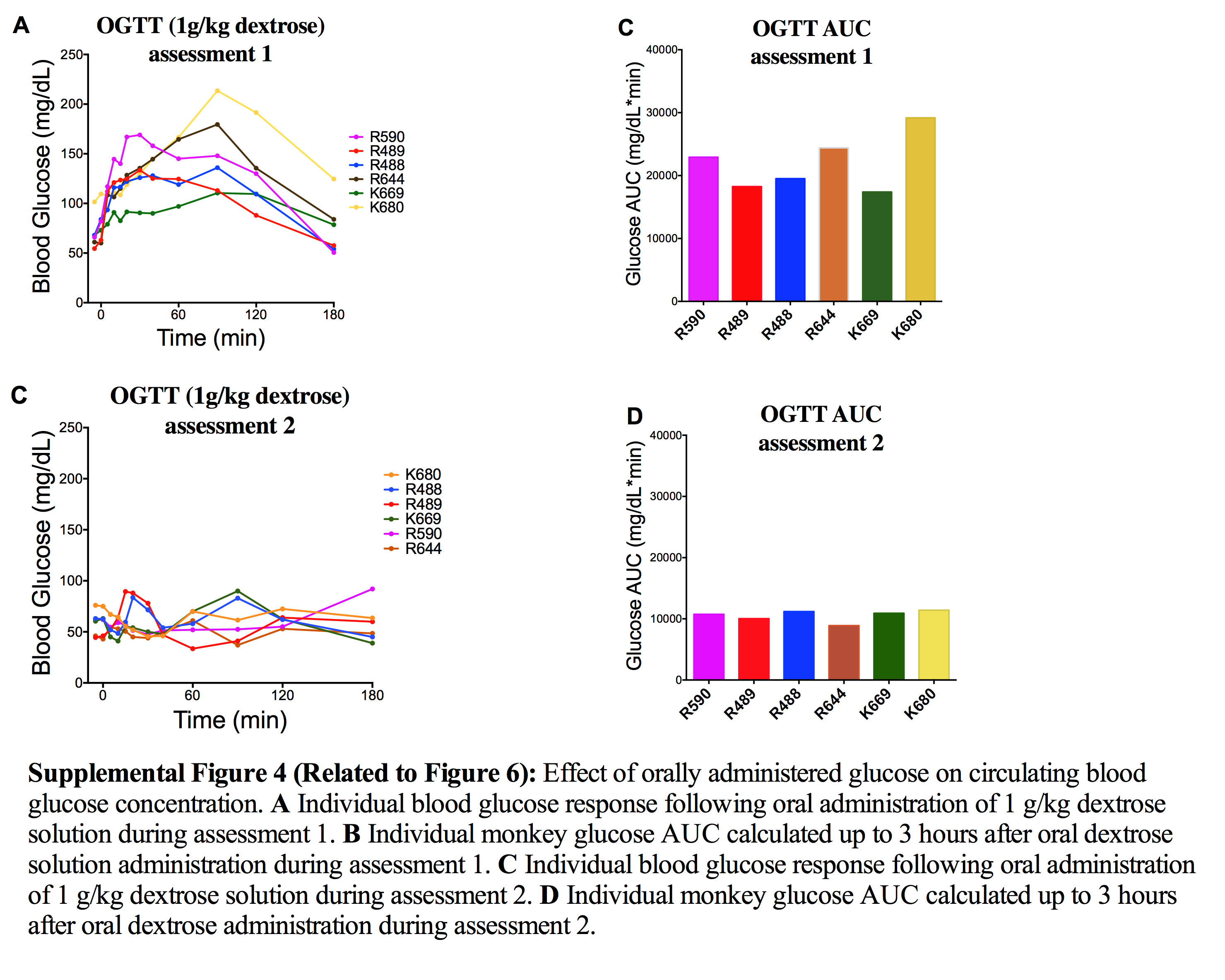

Supplement: Supplementary file 4 [file JMP-48-10-s004.tiff]
