# Supplementary material for: Characterization of glucose‐stimulated insulin release protocols in african green monkeys (Chlorocebus aethiops)
Source: J Med Primatol. 2018 Oct 25;48(1):10–21. doi: 10.1111/jmp.12374 (PMC6587791; doi:10.1111/jmp.12374)
Supplement: Supplementary file 5 [file JMP-48-10-s005.docx]

| **Monkey ID** | **Group** | **Sex** | **Age/ Estimated Age*** | **Weight** | **Girth** | **Crown-rump length** | **BMI** | **Cholesterol** | **HDL** | **LDL** | **Trigylcerides** |
| --- | --- | --- | --- | --- | --- | --- | --- | --- | --- | --- | --- |
|  |  |  | **(years)** | **(kg)** | **(cm)** | **(cm)** | **(kg/m^2^)** | **(mg/dl)** | **(mg/dl)** | **(mg/dl)** | **(mg/dl)** |
| B033 | Geriatric | F | 26 | 4.716 | 40 | 42 | 28 | 155 | 56 | 102 | 40 |
| B048 | Geriatric | F | 26 | 3.002 | 31 | 40 | 19 | 179 | 56 | 136 | 56 |
| E029 | Geriatric | F | 23 | 4.44 | 36.5 | 44 | 23 | 163 | 75 | 91 | 41 |
| F053 | Geriatric | F | 22 | 3.86 | 36 | 36 | 30 | 100 | 28 | 48 | 133 |
| H057 | Geriatric | F | 20 | 3.1 | 32.5 | 38 | 21 | 120 | 65 | 48 | 45 |
| I021 | Geriatric | F | 19 | 5.328 | 39 | 43 | 29 | 180 | 61 | 124 | 80 |
| **Mean Geriatric Females** | | | **22.7** | **4.1** | **35.8** | **40.5** | **25.0** | **149.5** | **56.8** | **91.5** | **65.8** |
| **Stdev Geriatric Females** | | | **2.9** | **0.9** | **3.5** | **3.1** | **4.6** | **32.7** | **15.8** | **37.2** | **36.1** |
| K330 | Geriatric | M | 16* | 6.076 | 35.5 | 46 | 29 | 135 | 87 | 43 | 34 |
| T964 | Geriatric | M | 22* | 6.41 | 39 | 46 | 30 | 160 | 74 | 87 | 53 |
| U461 | Geriatric | M | 22* | 5.036 | 34 | 43 | 27 | 126 | 65 | 58 | 49 |
| V068 | Geriatric | M | 19* | 5.688 | 34 | 45 | 28 | 142 | 57 | 87 | 32 |
| W733 | Geriatric | M | 20* | 6.494 | 40 | 47 | 29 | 123 | 66 | 53 | 44 |
| X587 | Geriatric | M | 14* | 6.44 | 39 | 47 | 29 | 137 | 71 | 68 | 34 |
| **Mean Geriatric Males** | | | **18.8** | **6.0** | **36.9** | **45.7** | **28.7** | **137.2** | **70.0** | **66.0** | **41.0** |
| **Stdev Geriatric Males** | | | **3.3** | **0.6** | **2.7** | **1.5** | **1.0** | **13.2** | **10.2** | **18.2** | **8.9** |
| R553 | Young | F | 6 | 4.036 | 29 | 41 | 24 | 128 | 50 | 79 | 48 |
| R582 | Young | F | 5 | 4.052 | 28 | 42 | 23 | 120 | 53 | 67 | 49 |
| R590 | Young | F | 5 | 4.45 | 34 | 40.5 | 27 | 158 | 74 | 86 | 26 |
| R613 | Young | F | 4 | 4.3 | 32.5 | 38 | 30 | 137 | 72 | 54 | 41 |
| R633 | Young | F | 4 | 4.908 | 35 | 41.5 | 28 | 113 | 67 | 39 | 29 |
| R646 | Young | F | 4 | 4.28 | 33 | 36 | 33 | 144 | 79 | 64 | 43 |
| **Mean Young Females** | | | **4.7** | **4.3** | **31.9** | **39.8** | **27.5** | **133.3** | **65.8** | **64.8** | **39.3** |
| **Stdev Young Females** | | | **0.8** | **0.3** | **2.8** | **2.3** | **3.7** | **16.5** | **11.8** | **17.0** | **9.7** |
| R485 | Young | M | 6 | 4.75 | 32 | 44 | 25 | 121 | 69 | 57 | 33 |
| R488 | Young | M | 6 | 7.238 | 38 | 47 | 33 | 150 | 88 | 61 | 28 |
| R489 | Young | M | 4 | 6.42 | 39 | 46 | 30 | 93 | 59 | 35 | 29 |
| R499 | Young | M | 6 | 7.504 | 40.5 | 47.5 | 33 | 185 | 98 | 53 | 22 |
| R644 | Young | M | 6 | 5.346 | 31 | 44 | 28 | 102 | 57 | 41 | 35 |
| R606 | Young | M | 4 | 6.666 | 35 | 47 | 30 | 147 | 76 | 47 | 20 |
| **Mean Young Males** | | | **5.3** | **6.3** | **35.9** | **45.9** | **29.8** | **133.0** | **74.5** | **49.0** | **27.8** |
| **Stdev Young Males** | | | **1.0** | **1.1** | **3.9** | **1.6** | **3.1** | **34.3** | **16.2** | **9.9** | **5.9** |

Supplemental Table 1: Baseline phenotypic data for individual geriatric and young monkeys

* Indicates age was estimated based on a combination of length in captivity, body weight when captured, dentation and general physical appearance.

BMI = body mass index; HDL = high density liproprotein; LDL = low density liproprotein
